# Incremental yield of exome sequencing over standard prenatal testing in structurally normal fetuses: systematic review and meta‐analysis

**DOI:** 10.1002/uog.29195

**Published:** 2025-02-17

**Authors:** A. Sotiriadis, E. Demertzidou, A. Ververi, E. Tsakmaki, C. Chatzakis, F. Mone

**Affiliations:** ^1^ Second Department of Obstetrics and Gynecology, Faculty of Medicine Aristotle University of Thessaloniki Thessaloniki Greece; ^2^ Department of Genetics for Rare Diseases Papageorgiou General Hospital Thessaloniki Greece; ^3^ Centre for Public Health, Queen's University Belfast UK

**Keywords:** exome sequence, fetus, meta‐analysis, monogenic, single‐gene disorder

## Abstract

**Objective:**

To critically review the literature and synthesize evidence on the incremental yield of prenatal exome sequencing (PES) in fetuses with an apparently normal phenotype with a normal G‐banded karyotype or chromosomal microarray (CMA).

**Methods:**

This systematic review and meta‐analysis was conducted using a predetermined protocol and registered with PROSPERO (ID: CRD42024593349). We included observational cohort studies reporting on the incremental yield of PES in fetuses with an apparently normal phenotype and a previously normal G‐banded karyotype/CMA. The risk of bias of the included studies was assessed using the Newcastle–Ottawa Scale. The pooled proportion of events was calculated using generalized linear mixed models, using the metaprop function in R version 2.15.1.

**Results:**

Four studies (1916 fetuses) were included in this systematic review and meta‐analysis, of which 32 cases had a pathogenic or likely pathogenic variant. The pooled incremental yield of PES in fetuses with an apparently normal phenotype was 1.6% (95% CI, 1.0–2.6%); the majority of variants were *de novo* within genes associated with autosomal dominant inherited conditions (pooled incremental yield, 0.9% (95% CI, 0.5–1.7%)). Based on the expected severity of the associated disease, the pooled incremental yield was 0.5% (95% CI, 0.1–1.5%) for severe disease and 0.5% (95% CI, 0.2–1.5%) for moderate disease. There were insufficient data to conduct the predefined secondary analyses according to normality of phenotype at birth, variants of uncertain significance and expected age of disease onset.

**Conclusion:**

Pooling data from four studies, we found that 1.6% of phenotypically normal fetuses with a normal G‐banded karyotype or CMA may have a pathogenic or likely pathogenic variant identified on PES, most of which occur *de novo*. The likelihood of a variant being associated with severe disease in such fetuses is 0.5%. However, more research is needed regarding the development of a universal classification of disease severity and the utilization of this evidence in clinical practice. © 2025 The Author(s). *Ultrasound in Obstetrics & Gynecology* published by John Wiley & Sons Ltd on behalf of International Society of Ultrasound in Obstetrics and Gynecology.

## INTRODUCTION

The prenatal application of exome sequencing has been a major development in prenatal genomic diagnosis. Thus far, the majority of research and clinical practice has focused on the application of prenatal exome sequencing (PES) in fetuses with a structural anomaly detected on prenatal ultrasound. Two major studies, the Prenatal Assessment of Genomes and Exomes (PAGE) study^1^ and the Columbia cohort study^2^, showed that the application of PES in fetuses with unselected anomalies and a normal G‐banded karyotype and/or normal chromosomal microarray (CMA) had an incremental yield of approximately 10% overall. Since then, further evidence suggests that the yield increases significantly when cases are preselected by a clinical geneticist and when applied to specific phenotypes (e.g. echogenic kidneys with a normal bladder; suspected skeletal dysplasia), where incremental yield is as high as 50–68%^3^, ^4^. This evidence has been applied in publicly funded health services, such as the National Health Service in England, with a view to balance diagnostic yield with cost and the proportion of variants of uncertain significance or secondary findings identified, if reported^5^. In non‐resource‐limited or privately funded health services, pregnant couples are often open to using any genomic test that is available prenatally and clinicians are obliged to advise them of such, hence it has become an emerging clinical practice in such countries to perform PES in fetuses without a detected structural anomaly^6^. Although there has been some literature reflecting on the ethical and interpretational challenges posed by such practice^7^, emerging evidence is yet to be combined to determine the pooled incremental yield of PES in the setting of structurally normal fetuses. Therefore, the aim of this systematic review and meta‐analysis was to critically review the literature and synthesize evidence on the incremental yield of exome sequencing in fetuses with an apparently normal phenotype.

## METHODS

This systematic review and meta‐analysis was conducted using a predetermined protocol, established according to the Cochrane Handbook's recommendations^8^. The systematic review adhered to the Preferred Reporting Items for Systematic Reviews and Meta‐Analyses (PRISMA) guidelines^9^ and was registered with PROSPERO (ID: CRD42024593349).

### Eligibility criteria

Studies eligible for inclusion were prospective or retrospective observational cohort studies reporting on the incremental yield of PES in fetuses with an apparently normal phenotype and absence of aneuploidy or copy‐number variants as determined by G‐banded karyotyping or CMA. Both singleton and twin pregnancies were eligible for inclusion. Only studies published in the English language were included. Studies in which monogenic disease was ascertained by methods other than PES (i.e. clinical examination) were excluded. Case reports and case series reporting on fewer than 10 fetuses were also excluded to avoid small‐study effects.

### Search strategy and study selection

PubMed, The Cochrane Library, Google Scholar and Scopus databases and the International HTA database and ClinicalTrials.gov registers were searched from 1 January 2018 to 25 September 2024 and repeated before submission of this study (when no additional studies were identified), for observational studies reporting on the incremental yield of PES in fetuses with an apparently normal phenotype. A combination of the following terms was used in the search: ‘exome‐’, ‘genome‐’, ‘sequencing’, ‘fetus’, ‘normal’, ‘uncomplicated’ and ‘routine’. Furthermore, an electronic search of the related articles (PubMed function) as well as a manual search of the reference lists of the selected articles was performed as an additional step to identify any potentially missed studies. All studies were compared to avoid inclusion of duplicates or overlapping samples and were screened based on their title and abstract. In cases of overlap, the study with the largest number of events was included. The authors of the primary studies were contacted in cases of missing data or for data clarification.

Two authors (E.D. and E.T.) assessed independently the eligibility of all identified citations according to the abovementioned criteria. After exclusion of ineligible studies, http://retractiondatabase.org was searched for any additional studies that may have been retracted. Disagreements between authors were resolved by consensus.

### Types of outcome measures

The main outcome was the incremental yield of exome sequencing, defined as the rate of pathogenic or likely pathogenic variants in fetuses with an apparently normal phenotype and absence of aneuploidy or copy‐number variants as determined by G‐banded karyotyping or CMA.

Additional outcomes that we intended to analyze were the pooled rates of: (1) pathogenic/likely pathogenic variants in fetuses with a postnatally proven normal phenotype (i.e. lack of major anomalies in postnatal examination); (2) pathogenic/likely pathogenic variants in apparently normal fetuses in which defects were diagnosed postnatally; (3) variants of unknown significance (VOUS); (4) pathogenic variants resulting in early‐onset disease; (5) pathogenic variants resulting in late‐onset disease; and (6) inheritance types of pathogenic variants. *Post‐hoc* subanalysis was performed according to severity of disease and origin and inheritance pattern of the variant(s). The severity of disease was reclassified where needed (for studies already reporting severity) or was primarily classified (for studies not reporting severity) by two clinical experts (F.M. and A.V.), according to Lazarin *et al*.^10^.

### Data extraction

Data extraction and assessment of study quality were performed independently by two authors (E.D. and C.C.). Extracted data included: details of authors; PMID/DOI; country; setting (i.e. public or private sector; clinical or research based); dates; eligibility criteria, with particular focus on how a ‘normal’ prenatal phenotype was defined in each study; gestational age at prenatal genomic testing; type of invasive tests used; type of previous genomic test (i.e. G‐banded karyotyping or CMA); type of next‐generation sequencing (NGS) ‐based test used; trio or fetus‐only sampling; attrition rate and causes; outcome of fetuses; postnatal ascertainment of prenatal phenotype or assessment for phenotypic evolution in pregnancy; specific genomic diagnosis for each of the pathogenic/likely pathogenic results and VOUS. The characteristics of each included study were assessed according to a predefined data extraction form included in the Cochrane Handbook for Systematic Reviews^8^. In cases of disagreement, a consensus was reached after discussion between the two authors. The pooled proportions of the outcomes of interest were calculated.

### Assessment of risk of bias

The risk of bias of the included studies was assessed independently by two authors (C.C. and E.T.) according to the Newcastle–Ottawa Scale (NOS). This scale was developed to assess the quality of observational (e.g. cohort, case–control, cross‐sectional) studies and non‐randomized clinical trials. Studies are judged on eight items, categorized into three groups: the selection of the study groups, the comparability of groups and the ascertainment of either the exposure or outcome of interest. A star is awarded for each item; the highest quality studies are awarded nine stars.

### Synthesis of results

The data from each study were extracted and the proportion of events for each outcome, with 95% CI, was estimated for each study. The pooled estimate was calculated using the metaprop function in an open‐source software R version 2.15.1 (The R Foundation for Statistical Computing, Vienna, Austria). Metaprop is used to perform meta‐analyses by implementing procedures that are specific to binomial data and that allow computation of exact binomial CI. A generalized linear mixed models (GLMM) approach was chosen over the inverse variance method. GLMM performs better when data are sparse and when proportions are close to extremes (0% or 100%). Additionally, with the GLMM approach, the weight of each study is not calculated and no continuity correction is required for studies with zero events^11^, ^12^. Given the anticipated heterogeneity due to the diversity in outcomes and diagnostic methods, the summary effect sizes were calculated using a random‐effects model, which assumes that the true effect size varies between the studies and that the included studies represent a random sample of effect sizes that could have been observed. We opted to use this model as it allows for variation both within and between studies, providing a conservative estimate of the summary statistics with wider CI. Pooled proportions were calculated using the GLMM approach and when at least two studies reported outcomes of interest, the forest plots were illustrated. The heterogeneity between studies was assessed using the *I* ^2^ statistic. A sensitivity analysis was planned to include only studies with fetuses with a postnatally confirmed normal phenotype.

## RESULTS

### Study selection

**Figure 1:**
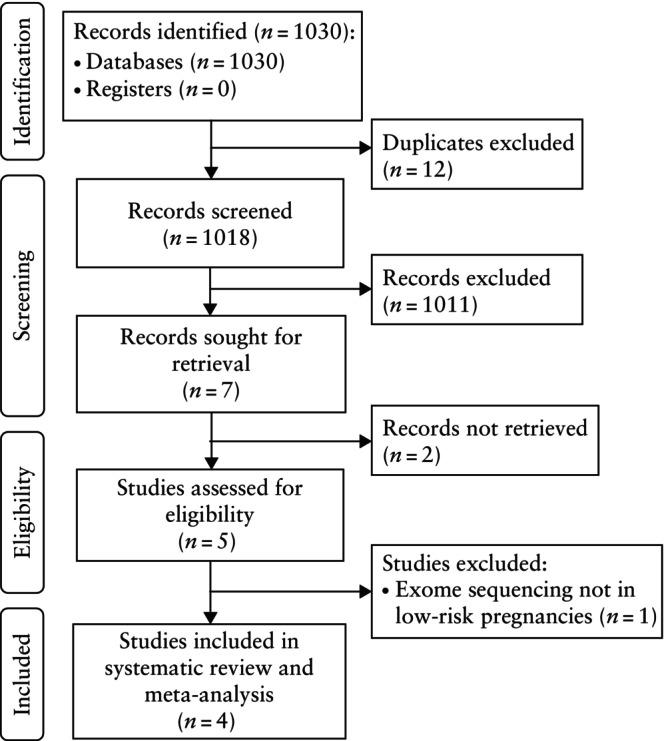
PRISMA flowchart summarizing search strategy and inclusion of studies in systematic review and meta‐analysis.

The electronic search yielded 1030 results. After exclusion of duplicates and exclusion of articles based on their title and/or abstract, seven articles remained for full‐text review. Five of these studies appeared eligible for inclusion^6^, ^13^, ^14^, ^15^, ^16^, of which four were ultimately included in the systematic review and meta‐analysis^6^, ^13^, ^14^, ^15^; the study of Akalin *et al*.^16^ was excluded because NGS‐based testing was performed only in fetuses with an abnormal phenotype (Figure 1).

### Study characteristics

The characteristics of the included studies are described in Table 1. All four studies were retrospective and used exome sequencing for genomic diagnosis after a normal CMA. All but one study performed trio analysis in all cases; Daum *et al*.^14^ only used trio analysis in approximately half of the cases.

**Table 1:** Characteristics of four studies included in systematic review and meta‐analysis

Study	Country	*n*	P/LP variants (*n*)	Inclusion criteria	Sampling	Exclusion criteria	Reason for parental request for NGS‐based testing
Daum (2023)^14^	Israel	482	4	No medical indication, parental request for ES, normal CMA, sonographically normal at the date of sampling	CVS or amniocentesis, GA not specified	Abnormal sonographic findings or first‐/second‐trimester testing, known parental illness, P/LP variant in CMA or CNV analysis in ES	85.7%, no specific reason; 10%, previous child with *de‐novo* pathogenic variant; 2.7%, increased paternal age; 1.6%, consanguinity
Gao (2024)^15^	China	254	6	No medical indication, parental request for ES, normal CMA	CVS, amniocentesis or cordocentesis at 13 + 4 to 30 + 3 weeks	Abnormal ultrasonographic findings	89.4%, previous child with monogenic disease and current pregnancy normal Sanger sequencing/MLPA; 7.1%, unspecified family concerns; 5%, previous pregnancy with *de‐novo* chromosomal disorder
Levy (2024)^6^	Israel	1020	21	Normal first‐ and second‐trimester prenatal tests, non‐consanguineous parents, normal CMA	Amniocentesis at 17–22 weeks	NT > 3 mm, major fetal structural anomaly, IUGR, polyhydramnios (AFI ≥ 25 cm), minor ultrasound scan findings (e.g. mildly echogenic bowel, absent DV, cardiac echogenic focus, abnormality in first or second biochemical tests, any abnormality in CMA, family history of monogenic disease, parents with same positive disease carrier status (identified prospectively) or parent with known dominant or X‐linked monogenic disease	Not specified
Vaknin (2022)^13^	Israel	160	1	Normal surveillance in pregnancy, non‐consanguineous parents, normal CMA (Subgroup (group 2a) of a cohort of 353 fetuses with non‐consanguineous parents with normal CMA and various ultrasonographic findings)	CVS or amniocentesis at 12–36 weeks	Group 2a*: Minor ultrasound findings that mildly increased risk for genetic disease, and all findings were not considered ‘out of range’ or ‘anomaly’ (e.g. mildly echogenic bowel, absent DV, femoral length between 5^th^ and 25^th^ centile, and head circumference between 5^th^ and 15^th^ centile). Group 2b†: Cases without any suspicious findings in pregnancy	Unspecified family concern

Only first author is given for each study.

* Included in meta‐analysis.

† Not included in meta‐analysis.

AFI, amniotic fluid index; CMA, chromosomal microarray; CNV, copy‐number variant; CVS, chorionic villous sample; DV, ductus venosus; ES, exome sequencing; GA, gestational age; IUGR, intrauterine growth restriction; LP, likely pathogenic; MLPA, multiplex ligation‐dependent probe amplification; NGS, next‐generation sequencing; NT, nuchal translucency; P, pathogenic.

All four studies included fetuses with normal ultrasonographic findings in first‐ and second‐trimester screening that did not present with any medical indication for genetic testing but were tested according to parental request. Vaknin *et al*.^13^ included these fetuses as part of a larger cohort of fetuses with various ultrasonographic findings, analyzing all the NGS results conducted in their population. Levy *et al*.^6^ did not report the reason for the parents' request. Vaknin *et al*.^13^ did not specify the family concern that drove their request. Daum *et al*.^14^ reported that the majority of parents (85.7%) did not present with a specific reason for their request, and of those that did, 10% of families had a previous pregnancy affected by a *de‐novo* pathogenic variant, 2.7% had a father of increased age and 1.6% were consanguineous parents. Gao *et al*.^15^ reported that the vast majority of parents (89.4%) already had an affected child with monogenic disease or a previous pregnancy with a *de‐novo* chromosomal defect (5%); few had unspecified family concerns (7.1%).

Levy *et al*.^6^ and Vaknin *et al*.^13^ reported the ultrasonographic exclusion criteria and explicitly excluded cases with consanguineous parents. None of the studies reported ultrasonographic ascertainment of ‘normality’ by the end of pregnancy or postnatal confirmation of normal phenotype. All four studies used the American College of Medical Genetics and Genomics (ACMG) guidelines to classify the variants as pathogenic or likely pathogenic^17^. In the study of Vaknin *et al*.^13^ the PM2 criterion was applied at a ‘supporting’ level of significance rather than a ‘moderate’ level of significance in accordance with the ClinGen recommendations, but the study did not align with the ClinGen recommendation for PP3 and BP4^18^, ^19^.

All included studies reported pathogenic and likely pathogenic variants. Only Levy *et al*.^6^ reported the identified cases of VOUS. The authors of the other three studies were contacted regarding VOUS, but no response was received. Regarding secondary findings, Daum *et al*.^14^ reported ACMG secondary findings ‘relevant to childhood’ and Gao *et al*.^15^ reported all ACMG secondary findings, not only those specifically with onset in childhood. The authors did not specify how these secondary findings were defined, as all fetuses were apparently unaffected and had no primary diagnosis. Levy *et al*.^6^ and Vaknin *et al*.^13^ reported secondary findings in parents who had consented and underwent trio testing, but not in the fetuses.

### Risk of bias

The NOS was used for the included cohort studies. All four were single‐arm cohort studies, therefore, NOS was modified accordingly to not include the ‘comparability’ section, thus setting the highest quality award as 6 stars. Three studies^6^, ^13^, ^14^ scored 6/6 stars and one study^15^ scored 5/6 stars; as a result, all studies were categorized as ‘low risk of bias’. The quality assessment of the included studies is presented in Table S1.

### Overall yield

**Figure 2:**
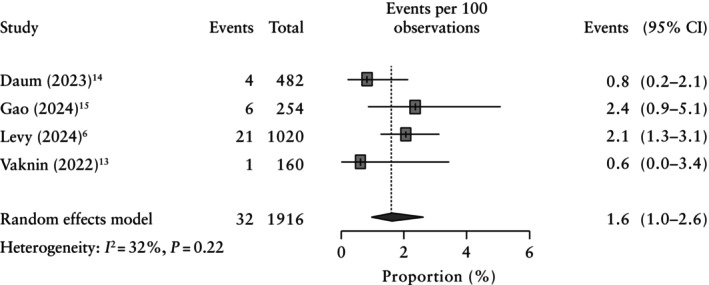
Forest plot showing pooled incremental yield of prenatal exome sequencing in fetuses with an apparently normal phenotype. Only first author is given for each study.

There were 32 individual cases with a pathogenic or likely pathogenic variant identified among the 1916 structurally normal fetuses. The characteristics of the included variants are highlighted in Tables 2 and 3. The incremental yield of PES in fetuses with an apparently normal phenotype was 1.6% (95% CI, 1.0–2.6%) (Figure 2).

**Table 2:** Characteristics of 32 identified pathogenic or likely pathogenic variants, according to study

Study/inheritance	Severity
Daum (2023)^14^ (*n* = 4 of 482)	
Autosomal recessive (*n* = 2)	
Compound heterozygous in *ATP7B*	Severe
Compound heterozygous in *NR2E3*	Moderate
Autosomal dominant (*n* = 2)	
*De novo* in *SPRED1*	Moderate*
*De novo* in *FGFR3*	Moderate
Gao (2024)^15^ (*n* = 6 of 254)	
Autosomal recessive (*n* = 2)	
Compound heterozygous in *GJB2*	Moderate†
Compound heterozygous in *NAGLU*	Profound†
Autosomal dominant (*n* = 4)	
*De novo* in *OPA1*	Moderate†
Maternally inherited in *PKD2*	Moderate†
Maternally inherited in *SDHB*	Moderate†
*De novo* in *NF1*	Moderate†
Levy (2024)^6^ (*n* = 21 of 1020)	
Autosomal recessive (*n* = 1)	
Compound heterozygous in *PAH*	Mild
Autosomal dominant (*n* = 19)	
*De novo* in *ADNP*	Severe
*De novo* in *SETD1A*	Severe
*De novo* in *PUM1*	Severe
*De novo* in *ANKRD11*	Severe
*De novo* in *TSC2*	Severe
*De novo* in *CUL3*	Severe
*De novo* in *SCN8A*	Severe
*De novo* in *WAC*	Severe
*De novo* in *KAT6A*	Severe
*De novo* in *EXT2*	Mild
Maternally inherited in *ENG*	Mild
Maternally inherited in *HARS*	Mild
Maternally inherited in *ACVRL1*	Mild
*De novo* in *COL11A1*	Mild
Maternally inherited in *TSHR*	Mild
Paternally inherited in *FBN1*	Moderate*
Paternally inherited in *SOS1*	Severe*
*De novo* in *COL4A1*	Mild
*De novo* in *NPR2*	Mild
X‐linked recessive (*n* = 1)	
Maternally inherited in *CACNA1F* to a male fetus	Moderate*
Vaknin (2022)^13^ (*n* = 1 of 160)	
Autosomal dominant (*n* = 1)	
*De novo* in *KCNK4*	Severe†

Only first author is given for each study.

* Severity reported and reclassified.

† Severity not reported and primarily classified.

**Table 3:** Class, origin, inheritance and severity of 32 identified pathogenic or likely pathogenic variants

Variable	*n* (%)
Class	
Likely pathogenic	26 (81.25)
Pathogenic	6 (18.75)
Origin	
*De novo*	18 (56.25)
Maternally inherited	7 (21.88)
Paternally inherited	2 (6.25)
Compound heterozygous	5 (15.63)
Inheritance	
Autosomal recessive	5 (15.63)
Autosomal dominant	26 (81.25)
X‐linked recessive	1 (3.13)
Severity	
Mild	9 (28.13)
Moderate	10 (31.25)
Severe	12 (37.50)
Profound	1 (3.12)

### Mode of inheritance

Of the 32 cases with a pathogenic or likely pathogenic variant, 26 were within autosomal dominant and five were within autosomal recessive disease‐causing genes, with a diagnostic yield of 1.1% (95% CI, 0.6–2.3%) and 0.3% (95% CI, 0.1–0.6%), respectively (Figures S1 and S2). The autosomal dominant conditions included 18 *de‐novo* variants and eight inherited variants (six maternally inherited and two paternally inherited). The diagnostic yield for *de‐novo* variants was 0.9% (95% CI, 0.5–1.7%) (Figure S3). Regarding inherited variants, Levy *et al*.^6^ included a fetus with a paternally inherited variant in the *FBN1* gene and reported that the father had typical features of Marfan syndrome. It was not specified whether the father was aware of his diagnosis. The other parents with identified autosomal dominant conditions were reported to be asymptomatic, including a father with a likely pathogenic variant in *SOS1*, causative of Noonan syndrome 4. Gao *et al*.^15^ included one mother with a likely pathogenic variant in *PKD2*, causative of polycystic kidney disease type 2, and another mother with a likely pathogenic variant in *SDHB*, causative of pheochromocytoma. All five fetuses with a variant in genes associated with autosomal recessive conditions had compound heterozygous variants. There were insufficient data to explore the diagnostic yield in X‐linked recessive inheritance, as there was only one case with a variant in a gene associated with an X‐linked recessive condition^6^ and no cases with a variant in a gene associated with an X‐linked dominant condition.

### Disease severity

**Figure 3:**
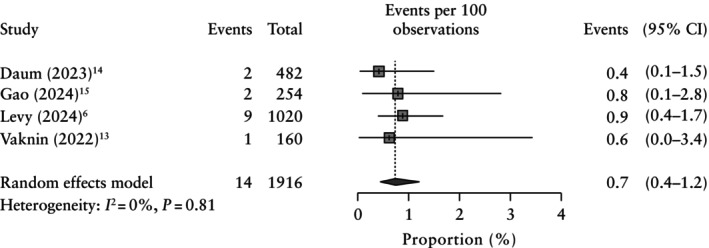
Forest plot showing pooled incremental yield of prenatal exome sequencing in fetuses with an apparently normal phenotype which had a *de‐novo* variant associated with moderate or severe disease, according to the Lazarin *et* *al*.^10^ severity classification. Only first author is given for each study.

A subgroup analysis according to disease severity showed that there were 12 variants associated with severe disease and 10 variants associated with moderate disease, with an incremental yield of 0.5% (95% CI, 0.1–1.5%) and 0.5% (95% CI, 0.2–1.5%), respectively (Figures S4 and S5). There were insufficient data to explore the incremental yield for variants associated with mild disease, as there were nine cases reported in only one study^6^, or profound disease, as there was only one case^15^. In total, 14 fetuses had a *de‐novo* variant associated with moderate or severe disease; the incremental yield of PES in this group of fetuses was 0.7% (95% CI, 0.4–1.2%) (Figure 3).

There were insufficient data to conduct predefined secondary analyses according to normality of phenotype at birth, VOUS and expected age of disease onset.

## DISCUSSION

After pooling data from the four studies on phenotypically normal fetuses, we found that the incremental yield of PES was 1.6%. The majority of pathogenic or likely pathogenic variants were monoallelic within genes associated with autosomal dominant inherited conditions (pooled yield, 1.1%), with the remainder of variants related to autosomal recessive (pooled yield, 0.3%) and X‐linked recessive inheritance. The incremental yield of PES in severe disease was 0.5% and for moderate disease it was also 0.5%. The majority of the variants were *de novo* (pooled yield, 0.9%), and the incremental yield for moderate or severe diseases caused by *de‐novo* variants was 0.7%.

As highlighted in the recent commentary of Chandler *et al*.^7^, ‘if the prior probability of a genetic disease is extremely low within the population, generally coupled with the absence of family history or phenotype, the chance of the fetus being affected is also very low’. The authors noted that on independent reanalysis of the variants from the study of Levy *et al*.^6^, over half of the pathogenic or likely pathogenic variants could be reclassified as VOUS, highlighting a need for unification and clarity of guidance around variant interpretation within rare disease‐causing genes^7^. To resolve some of the questions raised, it would be helpful if the original research groups reclassified the reported likely pathogenic variants after 24–36 months to estimate the prevalence of the truly pathogenic variants, based on evolving classification data and/or postnatal phenotypes in cases in which pregnancy was continued.

A further challenge arises through the fact that the ultimate postnatal phenotype or evidence of phenotypic evolution throughout pregnancy was not reported by any of the studies included in this meta‐analysis. This, combined with the likelihood of incomplete prenatal phenotyping, signifies that it is unlikely that the cohorts presented were all truly structurally normal fetuses^6^, ^20^.

All studies stated that where a previous fetus had a genetic disease, this was tested for in the index pregnancy before any such case could be included in the study population. Nevertheless, parentally inherited conditions included Noonan syndrome 4 and Marfan syndrome, which are typically related to highly specific phenotypes, thus allowing for *Gestalt* diagnoses. In both of these cases, the fathers had apparently consented to genetic testing and provided blood samples, implying that they had been physically present at one consultation at least. This raises questions regarding the depth and extent of the family history recorded before characterizing these fetuses as ‘low risk’.

Gao *et al*.^15^ chose to include two fetuses with secondary findings among their positive cases. Both conditions were typically adult onset: polycystic kidney disease and pheochromocytoma/paraganglioma syndrome‐4, which, furthermore, exhibit incomplete penetrance. The mothers of the two fetuses were carriers of the variants and were unaware of this before their participation in the study. Polycystic kidney disease is not included in the latest version of the ACMG secondary findings list^21^. On the other hand, Daum *et al*.^14^ chose not to include among their primary diagnostic cases a known pathogenic variant in the *RET* gene associated with multiple endocrine neoplasia type IIA, instead reporting it as a secondary finding. The variant was paternally inherited but there was no recorded phenotype in the father or his family. Multiple endocrine neoplasia type IIA/B is included in the ACMG secondary findings list and can have an early onset, hence the recommendation for surveillance and intervention from the first year after birth^22^. Had the case of polycystic kidney disease been excluded as a primary diagnosis and the case of multiple endocrine neoplasia type IIA been included as a primary diagnosis in the meta‐analysis, the incremental yield would have remained at 1.6%. The reporting of secondary findings, specifically ones without childhood onset or incomplete penetrance, is highly controversial in terms of limiting the subsequent child's future autonomy, as well as the potential to cause undue stress and anxiety in the expectant couple^23^.

Despite the above concerns, an incremental yield of 1.6%, if indeed the true incremental yield, could be argued to be of significance, particularly if associated with a severe phenotype. The incremental yield of moderate or severe conditions caused by *de‐novo* variants was 0.7%. Moreover, three of the four studies were conducted in Israel, where preconception carrier screening is conducted routinely, meaning that common recessive conditions had been, to some extent, already excluded in these fetuses^6^, ^13^, ^14^.

The evidence that we pooled had several limitations. First, the overall evidence (*n* = 1916) is limited in size. Second, although all included studies examined phenotypically normal fetuses, the reasons for parental request for PES differed, potentially inducing selection bias. Third, conditions that do not technically constitute a defect but might increase the risk of a genomic anomaly (e.g. fetal growth restriction) were not explicitly excluded. Fourth, none of the studies reported confirmation of a normal phenotype at birth or later in pregnancy, which prevented us from being able to perform the corresponding prespecified subgroup analyses. Finally, most of the studies were performed in Israel, where healthcare professionals are obligated to inform pregnant patients of all globally available genetic tests, regardless of the indication, which may limit the generalizability of findings^7^.

Given the well‐described limitations of meta‐analyses of prenatal genomic investigation^24^, we sought to reduce publication bias by only including large series and by defining clearly incremental yield. Additionally, we aimed to address the problem of incomplete fetal phenotype by specifying the criteria by which phenotypic normality was defined in the primary studies, and report the pooled rate of VOUS. We contacted the authors of the primary studies asking for further information regarding these details; unfortunately, no further information could be retrieved.

We present only four small studies for this meta‐analysis and are aware that there is ongoing larger‐scale research being performed internationally, begging the question as to whether a systematic review is somewhat premature. Given the clinical and ethical implications of offering PES in phenotypically normal fetuses, as alluded to previously^7^, it is important to provide clinicians with a baseline pooled yield upon which to counsel patients, given that this practice is already occurring in developed countries such as Israel and the USA. It is anticipated that, with the publication of larger studies, the aforementioned issues regarding postnatal follow‐up and penetrance may be addressed further, combined with an evaluation of cost‐effectiveness and qualitative assessment of stakeholder views, which is yet to be determined for PES in fetuses with an apparently normal phenotype. The incremental yield of CMA over G‐banded karyotyping is 1% in structurally normal fetuses and 6% in structurally abnormal fetuses^25^. Although modest, it forms part of routine clinical practice in several countries internationally. Indeed, the same could be said of whole‐genome sequencing over PES, for which the incremental yield is 1% based on limited available studies^26^. Despite this, there is still a move towards adoption of a whole‐genome approach in several countries such as the UK and Sweden^27^.

To conclude, after pooling data from four studies we found that 1.6% of phenotypically normal fetuses with a normal CMA or karyotype may have a pathogenic or likely pathogenic variant identified using PES, most of which occur *de novo*. The likelihood of a variant being associated with severe disease in such fetuses is 0.5%. However, more research is needed regarding the development of a universal classification of disease severity and the utilization of this evidence in clinical practice.

## Supporting information


**Table S1** Risk of bias of included studies

**Figure S1** Forest plot showing pooled proportions of autosomal dominant variants.

**Figure S2** Forest plot showing pooled proportions of autosomal recessive variants.

**Figure S3** Forest plot showing pooled proportions of *de‐novo* variants.

**Figure S4** Forest plot showing pooled proportions of variants associated with severe disease.

**Figure S5** Forest plot showing pooled proportions of variants associated with moderate disease.

## Data Availability

The data that support the findings of this study are available from the corresponding author upon reasonable request.
