# Supplementary material for: Incremental yield of exome sequencing over standard prenatal testing in structurally normal fetuses: systematic review and meta‐analysis
Source: Ultrasound Obstet Gynecol. 2025 Feb 17;65(5):552–9. doi: 10.1002/uog.29195 (PMC12047680; doi:10.1002/uog.29195)
Supplement: Supplementary file 1 — Table S1 Risk of bias of included studies [file UOG-65-552-s002.docx]

**Table S1** Risk of bias of included studies

| Study | Selection | | | | ^e^Comparability | Outcome | | | Overall |
| --- | --- | --- | --- | --- | --- | --- | --- | --- | --- |
|  | ^a^ Representativeness  of the exposure  (intervention) cohort | ^b^ Selection  of the  nonexposed  cohort | ^c^ Ascertainment  of exposure | ^d^ Outcome of interest not present at start of study |  | ^f^Assessment  of outcome | ^g^ Length of follow up | ^h^ Adequacy of  follow up |  |
| Daum (2023)^14^ | B | NA | A | A | NA | B | A | A | Low risk |
| Gao (2024)^15^ | B | NA | A | A | NA | B | A | D | Low risk |
| Levy (2024)^6^ | B | NA | A | A | NA | B | A | A | Low risk |
| Vaknin (2022)^13^ | B | NA | A | A | NA | B | A | A | Low risk |

Only first author is given for each study.

1. ^a^A, truly representative of the average pregnant woman without PCOS; B, somewhat representative of the average pregnant woman without PCOS; C, selected group; D, no description of the derivation of the cohort.
2. ^b^A, drawn from the same source as the intervention cohort (concurrent controls); B, drawn from a different source (historical controls); C, no description of the derivation of the nonexposed cohort.
3. ^c^A, secure record (e.g., hospital records); B, structured interview; C, written self-report; D, no description.
4. ^d^Demonstration that outcome of interest was not present at the start of the study: A, yes; B, no.
5. ^e^Comparability of cohorts on the basis of the design or analysis: A, study controls for the most important factor; B, study controls for any additional factor; C, not carried out or not reported.
6. ^f^A, independent blind assessment; B, record linkage; C, self-report; D, no description.
7. ^g^Was follow-up long enough for outcomes to occur? A, yes; B, no.
8. ^h^A, complete follow-up; all subjects were accounted for. B, Subjects lost to follow-up were unlikely to introduce bias because small numbers were lost; >90% had follow-up, or description was provided of those lost. C, follow-up rate <90%, and there was no description of those lost. D, no statement.
